# A Foreign Speech Accent in a Case of Conversion Disorder

**DOI:** 10.1155/2005/989602

**Published:** 2006-02-27

**Authors:** Jo Verhoeven, Peter Mariën, Sebastiaan Engelborghs, Hugo D’Haenen, Peter De Deyn

**Affiliations:** ^1^Department of LinguisticsUniversity of AntwerpAntwerpBelgium; ^2^Department of LanguagesVrije Universiteit BrusselBrusselsBelgium; ^3^Department of NeurologyZNA-Middelheim HospitalAntwerpBelgium; ^4^Laboratory of Neurochemistry and BehaviourBorn-Bunge FoundationUniversity of AntwerpAntwerpBelgium; ^5^Department of PsychiatryAcademic HospitalVrije Universiteit BrusselBrusselsBelgium

**Keywords:** Foreign accent syndrome, conversion disorder, neurolinguistics

## Abstract

Objective: The aim of this paper is to report the psychiatric, neuroradiological and linguistic characteristics in a native speaker of Dutch who developed speech symptoms which strongly resemble Foreign Accent Syndrome. Background: Foreign Accent Syndrome is a rare speech production disorder in which the speech of a patient is perceived as foreign by speakers of the same speech community. This syndrome is generally related to focal brain damage. Only in few reported cases the Foreign Accent Syndrome is assumed to be of psychogenic and/or psychotic origin. Method: In addition to clinical and neuroradiological examinations, an extensive test battery of standardized neuropsychological and neurolinguistic investigations was carried out. Two samples of the patient's spontaneous speech were analysed and compared to a 500,000-words reference corpus of 160 normal native speakers of Dutch. Results: The patient had a prominent French accent in her pronunciation of Dutch. This accent had persisted over the past eight years and has become progressively stronger. The foreign qualities of her speech did not only relate to pronunciation, but also to the lexicon, syntax and pragmatics. Structural as well as functional neuroimaging did not reveal evidence that could account for the behavioural symptoms. By contrast psychological investigations indicated conversion disorder. Conclusions: To the best of our knowledge this is the first reported case of a foreign accent like syndrome in conversion disorder.

## 1. Introduction

Foreign Accent Syndrome is a rare speech production disorder in which a patient's pronunciation is regarded as foreign by speakers of the same speech community. In most of the reported cases Foreign Accent Syndrome originates from focal brain damage in the precentral gyrus of the language-dominant hemisphere either as a result of trauma or stroke. The first case was reported by Pick [6] in 1919 who described a native speaker of Czech developing a Polish accent after a left hemisphere stroke. The impression of a foreign accent was mainly caused by wrong word stress placement and the softer pronunciation of Czech fricatives. The relatively few cases of foreign accent that have been described since then included patients who developed a German [9,16], Swedish [26], Welsh [34], French [25,31,38,49], Chinese [19], English [3,15,21,46], Slavic [28,33], Hungarian [33], Northern Irish [17], Korean [52], Dutch [12], Scottish [23], Nordic [41] and Spanish [18] accent. In a number of cases, the foreign accent was not unambiguously related to one single language but could be regarded as generic in nature [22,24,32,40,47,50]. It is interesting to note that in most cases, the native language of the patients was American English.

Only in a few cases the patient's foreign accent could not be directly linked to brain damage. Critchley [34]

ISSN 0953-4180/05/$17.00 © 2005 – IOS Press and the authors. All rights reserved described a 49-year-old patient suffering from post-traumatic neurosis after a car crash. Her speech was described as "curiously slow, syllabic and hesitant (...) with an excessive range of modulation, which was not wholly appropriate to the content of what she was saying". Her accent was identified by most people as Welsh. Reeves and Norton [46] reported the case of a 65-year-old man with a long history of schizophrenia being admitted with psychotic symptoms such as paranoid delusions, bizarre behaviour and auditory hallucinations. During these exacerbations he took on a British English accent that disappeared after administering risperidone. Gurd et al. [25] reported the case of an English-speaking 47-year-old female patient who developed a French accent after a sudden onset of aphasia and headache. The French accent was mainly caused by the abnormal syllable-timed speech rhythm and the effortful pronunciation of /h/. In addition, the patient's pitch level was exceptionally high, which contributed to the general foreign quality of her speech. The neurological picture was unclear, but there was doubt as to the organical cause of the speech symptoms. Van Borsel et al. [28] describe the case of a 32-year-old Dutch speaking woman who developed a foreign accent that was assessed by many of the participants in a listening panel as Eastern European. The predominant articulation errors were the devoicing of voiced consonants, cluster reduction of r-clusters, initial deletion of /h/ and final consonant deletion involving /r/. An analysis of the patient's speech prosody revealed errors in the placement of word and sentence stress and a tendency towards scanning speech. Van Borsel et al. [28] indicate that these speech symptoms are likely to be of psychogenic origin in view of the absence of a brain lesion, the history of psychiatric problems, the sudden onset and the bizareness of the speech patterns, the absence of struggle behaviour and the reversibility of the symptoms.

From the previous section, it can be concluded that Foreign Accent Syndrome in some case may be linked to psychiatric problems. Although a variety of psychogenic speech and language characteristics have been reported [1], foreign accent qualities have to the best of our knowledge never been described in conversion disorder. This paper describes the psychiatric, neuroradiological, neuropsychological and neurolinguistic findings in a Dutch patient who developed a French foreign accent a few hours after almost having been involved in a traffic incident as a cyclist. At the time of admission to our hospital, the foreign accent had persisted for 8 years.

## 2. Case description

In 2003, a 51-year-old right-handed woman was referred to our hospital because a Foreign Accent Syndrome of psychotraumatic origin was suspected. At the time of admission to the hospital the patient had been suffering from disrupted speech and progressive gait disturbances since 1995. She was wheelchair-bound. Medical antecedents were not contributory and no history of developmental or psychiatric disorder was found. She was a qualified teacher of Dutch who had been teaching Dutch as a foreign language in a French company for several years.

Neurobehavioural symptoms started in 1995 just after the patient had almost been involved in an accident with a car while riding her bicycle. After getting home she started behaving strangely and was confused. She developed a tremor in both hands and spoke in a stutter-like manner with what was perceived as a French accent by the members of her family. Two months after the incident the patient was assessed neurologically for the first time: a computerized tomography (CT) scan of the brain and an EEG were normal. Neurolinguistic investigations formally excluded aphasia, dysarthria or apraxia of speech. Despite intensive speech therapy, physiotherapeutic treatment and psychotherapy there had been no improvement of neurobehavioural symptoms.

## 3. Psychodiagnostic assessment

In 1995, the patient's personality was assessed by means of the Minnesota Multiphasic Personality Inventory (MMPI) [48] and the results matched the profile of a conversion disorder. The MMPI profile showed a Conversion V pattern. The complete configuration indicated preoccupation with health, denial of anxiety and unsteadiness. In addition, the patient showed a strong motive to please others, to seek social contacts but an insensitivity to the psychological needs of others.

In 2003, the MMPI and the dissociation Questionnaire (DIS-Q) [27] were administered. The patient obtained a total score of 1.4 which is near the mean score of 1.5 in a normal population.

## 4. Neurological characteristics

On admission to our hospital in 2003, a standard clinical neurological examination revealed a bizarre, inconsistent, wide-based and unsteady gait whereas co-ordination, muscle tone and tendon reflexes were normal. Structural brain imaging by means of Computerized Tomography and Magnetic Resonance Imaging of the brain did not disclose any abnormalities. A standard EEG, routine laboratory studies, a lumbar puncture with analysis of cerebrospinal fluid and serum vitamin B12 and red cell folate levels did not reveal anything abnormal.

## 5. Neuropsychological and neurolinguistic characteristics

During hospitalisation standardized neuropsychological and neurolinguistic tests were carried out. The neuropsychological test battery consisted of the Mini Mental State Examination [36], the Wechsler Adult Intelligence Scale [10], the Wechsler Memory Scale-Revised [11], parts of the Alzheimer's Disease Assessment Scale [51], subtests of the Hierarchic Dementia Scale [37], the Stroop Colour-Word test [8,35], the Trail Making Test [2,7], the Rey-Osterrieth Figure [42] and the Judgment of Line Orientation test [4]. Handedness was formally assessed by means of the Edinburgh Inventory [45]. The neurolinguistic test battery consisted of the Aachener Aphasie Test [43], a selection of subtests of a Dutch version of the Boston Diagnostic Aphasia Examination [20], the Boston Naming Test [13,44] and a semantic and phonological verbal fluency task (1 minute generation of animal names, means of transport, vegetables, clothes and words starting with phoneme F, A and S) (unpublished norms). On all of these tests, the patient obtained above average results.

## 6. Language characteristics

### *6.1. Language samples*

Two samples of the patient's language were investigated. The first sample was a spontaneous conversation between a neuropsychologist and the patient recorded in 1995, that is three months after the acute onset of symptoms. The second speech sample was a spontaneous conversation with two authors of this paper in 2003. In both interviews, the patient described what happened to her during and after the incident and spoke about various issues related to her personal and professional life. In terms of the topics discussed, the two speech samples can be regarded equivalent. Both samples consisted of the first 1,000 words of the conversation and covered approximately 15 minutes of speech.

In the analysis, some of the patient's speech characteristics were compared to a 500,000 word reference corpus of spontaneous speech from 160 native Dutch speakers from Belgium and The Netherlands. This corpus was available at the University of Antwerp: it had been collected for purposes unrelated to this research. The speakers in the corpus were all teachers of Dutch and their professional background was thus comparable to that of the patient. A more detailed description of the corpus is found in [30].

All the analyses were carried out by the first author of this paper who is an experienced phonetician/linguist. At a later stage, the same data were analyzed independently of this investigation in the context of an MA-dissertation project [14]. A comparison between both analyses did not reveal significant discrepancies.

### *6.2. General impressions*

The patient's speech can generally be characterized as very hesitant, mostly effortful with stretches of fluent and clear speech. Throughout, there is a strong impression of a French accent that is mainly caused by pronunciation errors, wordfinding problems, the occasional use of French words and syntactic structures that are very consistent with mistakes made by French learners/speakers of Dutch.

### *6.3. Lexicon*

At the lexical level, both speech samples are characterized by occasional language mixing and code switching. The patient sometimes uses French words (in 1995: 4 occurrences in the 1,000 words speech sample; in 2003: 8 occurrences in the 1,000 word speech sample). In addition, she uses French interjections such as 'oui', 'bon', 'hein' (1995: 1; 2003: 6) and applies strategies that foreign language learners typically use to compensate for wordfinding problems; that is, direct translations from French (1995: 0; 2003: 1), the adaptation of French words to the Dutch morphological system (1995: 0; 2003: 3), the paraphrasing of words (1995: 2; 2003: 1) and the use of neologisms (1995: 2; 2003: 0). Also very characteristic of the patient's speech is the extremely frequent use of pauses filled by words like 'Erhm'. In the 1995 sample 7.97% of the total number of pauses were filled. In 2003 this number has almost doubled to 16.41%. This value is almost 4 times higher than the use of filled pauses in normal

Table 1
Distribution of the different word classes in the 1995 and 2003 speech samples and the reference corpus

| Word class | 1995 | 2003 | Corpus |
|---|---|---|---|
| Nouns | 13.6% | 14.7% | 11.9% |
| Adjectives | 4.8% | 5.2% | 7.9% |
| Adverbs | 11.2% | 11.6% | 7% |
| Auxiliaries | 9.2% | 7.7% | 8% |
| Verbs | 13.4% | 15.7% | 15.2% |
| Personal pronouns | 14.5% | 12.8% | 18.3% |
| Possessive pronouns | 1.5% | 1% | 3% |
| Demonstrative pronouns | 1.3% | 1% | 1.5% |
| Interjections | 2.1% | 2.6% | 1% |
| Conjunctions | 6.1% | 7% | 7% |
| Prepositions | 9% | 6.2% | 8.6% |
| Definite articles | 6% | 5.2% | 5.6% |
| Indefinite articles | 1.7% | 1.4% | 2% |
| Other | 5.6% | 7.9% | 3% |

$\chi^2 = 9.40$, d.f. $= 26$, $p > 0.950$.

speakers of Standard Dutch in the reference corpus, i.e. 3.87%.

An analysis of the frequencies of the various word categories in the patient's speech revealed no significant differences between the 1995 and 2003 speech samples and they follow the normal distribution patterns for Dutch that were established in the reference corpus. The proportions for the different word categories are summarized in Table 1.

In addition to the distribution of the different word classes, the type/token ratio of content words (TTRC) was calculated for both the patient's speech samples. The type/token ratio is defined as the proportion of unique content words over the total number of content words. Content words were nouns, adjectives, verbs and adverbs. This ratio can be regarded as an indication of the diversity in the patient's content word vocabulary in spontaneous speech. For the 1995 speech sample the TTRC was 0.79 while that for the 2003 speech sample amounted to 0.86. The TTRC for her peer group in the Dutch reference corpus was 0.80. From this it can be concluded that the patient's content word vocabulary is entirely in agreement with what can be expected from her peers.

Finally, word complexity was investigated and expressed as the average number of syllables per word. In 1995, this value amounted to 1.3599 syll/word (range $=$ 5), while in 2003 it was numerically slightly higher with 1.4247 syll/word (range $=$ 5). A t-test indicates that this difference is not significant (F(1,1998) $= 0.8489$, $p = 0.3570$). These values compare extremely well with that of the reference corpus, which was established at 1.41 syllables per word (range $=$ 9).

### *6.4. Syntax*

The syntactic problems in the patient's speech are mainly related to word order. Without any exception, all the grammatical errors observed in the patient's speech are prototypical of French learners of Dutch.[1] These grammatical errors included mistakes against: sentence word order (1995: 34; 2003: 35), subject-verb correspondences (1995: 1; 2003: 2), the use of definite articles (1995: 6; 2003: 3), adjective-noun correspondence (1995: 2; 2003: 1). Each of these error types are exemplified in examples (1)-(4) respectively:

(1) 'En in het begin **ik heb** gehad . . .' instead of 'En in het begin heb ik gehad . . .' (*And in the beginning I had . . .*).
(2) 'Dan **gaat ik** naar de psychiater' instead of 'Dan **ga ik** naar de psychiater' (*Then I went to the psychiatrist*)
(3) '**De** lopen ging steeds minder' instead of '**Het** lopen ging steeds minder' (*Walking became more difficult*)
(4) 'Ik heb een **Latijnse temperament**' instead of 'Ik heb een **Latijns temperament**' (*I have a Latin temperament*).

### *6.5. Pragmatics*

It may be interesting to mention that in both speech samples the patient uses one or two self-corrections in which she dwells pragmatically on her status as a non-native speaker of Dutch. In example (5), she corrects a verb, in (6) the use of a personal pronoun. This is a phenomenon that we have regularly witnessed ourselves in real learners of Dutch when they show that they are consciously trying to stay in control of the correctness of their speech output.

(5) Heb mijn moeder gehaald . . . nee, OPgehaald (*Have my mother collected, . . . no, picked her up*)
(6) Zo, dat is mij . . . DAT BEN IK (giggle, emphatic) (*Right that is me . . . that is I*)

These two examples of metalinguistic reflection show that the patient portrays herself as a competent, linguistically aware non-native speaker of Dutch.

[1] This claim is based on the first author's extensive experience in foreign language teaching, specifically to French learners of Dutch and English.

### 6.6. Pronunciation

The patient's speech is characterized by a very prominent French accent. This impression of a French accent is mainly caused by the use of the French uvular-r: before developing the foreign accent, the patient used a vocalic-r that is very common in the Netherlands. In addition, we observed cumbersome articulation of the velar fricatives [x] and [$\gamma$], the excessively front articulation of the open back vowel [$\alpha$] and the too close articulation of the half-open unrounded front vowel [I]. In quite a number of cases, the patient makes pronunciation mistakes that are prototypical of French learners of Dutch, i.e. the extremely frequent wrong placement of word stress on the last syllable of words (1995: 40; 2003: 41), regular attempts to make wordfinal fricatives and stops voiced by adding [$\partial$] (1995: 21; 2003: 22) and the occasional strengthening of [x] and [$\gamma$] into [k] and [g] (1995: 2; 2003: 6).

Other aspects of the patient's speech do not contribute to the French accent, but have to be qualified as generally inappropriate. In the first instance, the patient's speech is characterized by a very strong creaky voice that was not present in the 1995 speech sample. In addition, both speech samples have a very slow speech rate[2]: expressed as the number of syllables per second the patient's speech rate in 1995 was 2.72 syll/sec. The patient's rate in 2003 had gone down a little to 2.25 syll/sec. The normal speech rate for Dutch ranges between 3.98 and 4.23 syllables per second [30].

Furthermore, the patient displays regular stutter-like behaviour both word-initially and word-finally (1995: 10; 2003: 50). Aside from iterations (anything up to 15 repetitions), no blocks or prolongations were found. Finally, inappropriate pausing in the middle of speech segments was regularly observed, particularly in stops (1995: 2; 2003: 14).

### 6.7. Comparison to patient's English and French

As part of the interviews conducted in 2003, some parts of the conversation with the patient were conducted in English and French. The patient's English was also characterized by a strong French accent: this was caused by the use of uvular-r and wrong word stress-placement on the last syllables of words. The patient's speaking rate in English was 2.26 syll/sec, which is comparable to her speech tempo in Dutch. Quite remarkably there were hardly any iterations and inappropriate pauses. In addition, no obvious syntactic problems were found in her English. The patient's French was actually the best language of all: her speaking rate is considerably faster than in the other languages (3.20 syll/sec) and there are no indications of a foreign accent nor any obvious syntactic problems.

## 7. Discussion and conclusion

The patient described in this paper is a native speaker of Dutch who developed a strong French accent after the traumatic experience of nearly having been involved in a traffic accident. Although the patient did not sustain physical injury in this accident, the psychological impact must have been severe. The French accent originates from a combination of lexical, grammatical and pronunciation characteristics. At the lexical level the patient shows occasional language mixing and code switching. In addition, she compensates word-finding problems by strategies that are typical for foreign language learners, i.e. direct translations from French, the adaptation of French words to the Dutch morphological system, the paraphrasing of words and the creation of neologisms. The Type/Token ratio suggests that the patient's content word vocabulary is entirely in agreement with her professional background.

At the grammatical level, the patient regularly makes mistakes that are characteristic of French learners of Dutch, particularly with respect to word order, subject-verb agreement and the use of definite articles before nouns. It should be pointed out that these inconsistent grammatical mistakes are very unlike the agrammatic phenomena observed in aphasic patients.

In addition, the patient did not make any grammatical errors in written language tasks or while speaking English. Most likely the patient is not familiar with the grammatical mistakes that are typically made by French learners of English.

[2]In the calculation of speech rate, a distinction is traditionally made between speaking rate and articulation rate. The former is expressed as the number of syllables per second and includes both silent and filled pauses (words as Erhm etc). The latter is also expressed as the number of syllables per second, but the measure excludes silent intervals. The measure of speech rate reported here refers to articulation rate. These measures were calculated by counting the number of syllables per utterance, while utterance duration was very accurately measured in a signal processing program PRAAT. This procedure was applied to each utterance and the values reported here are mean values averaged over all utterances.

In terms of pronunciation, the patient incorporated a few typical French pronunciation features such as the uvular-r, the excessively front articulation of the vowel [a] and pronunciation of [I] as [i]. Besides, the patient regularly makes mistakes that are again typical for French learners of Dutch such as wrong accent placement.

It is important to note that the speech symptoms do not seem to have an organic basis: all neurological investigations failed to reveal any evidence of an organic brain dysfunction. The psychiatric assessment of the patient, however, supported the diagnosis of a conversion disorder. The diagnostic criteria for conversion disorder include essentially:

– One or more symptoms or deficits affecting voluntary motor or sensory function that suggest a neurological or other general medical condition.
– Psychological factors are judged to be associated with the symptom or deficit because the initiation or exacerbation of the symptom or deficit is preceded by conflicts or other stressors.
– The symptom or deficit is not intentionally produced or feigned.

The emergence of a French accent in the patient's speech is reminiscent of what has been called Foreign Accent Syndrome. It should be pointed out, however, that there are important differences between the speech characteristics of Foreign Accent Syndrome in the literature and the speech output of this patient. In the first instance, the impression of foreign accent in Foreign Accent Syndrome is without exception created by the patient's pronunciation only. In this patient, however, the foreign accent originates from both speech and language characteristics. In this sense, the patient's speech output resembles that of a patient reported in Van Borsel et al. [28] who also report a number of language problems such as the dropping of articles, prepositions and auxiliaries. In addition, there were occasional grammatical errors involving plural formation of nouns, incorrect verb conjugations and incorrect word order.

Secondly, there is the degree of familiarity with the foreign accent. Patients with Foreign Accent Syndrome mostly do not have any previous experience with the foreign language the accent of which has emerged in their speech. This patient, however, used to be a teacher of Dutch as a foreign language and has taught Dutch to French learners. As such she is very familiar with the typical pronunciation/language mistakes that are made by these learners.

Thirdly, real Foreign Accent Syndrome gradually improves (slightly or disappears completely) over the years, i.e. in most of the reported cases it becomes less distinctly foreign. In this patient, however, the figures on a variety of dimensions suggest that the foreign accent and the degree of abnormality get progressively stronger.

Fourthly, in most reported cases Foreign Accent Syndrome results from a structural brain lesion involving the precentral area of the language dominant hemisphere. Structural damage to this region as well as indications for an organic expressive language disorder were formally ruled out.

This foreign accent, however, seems to be the consequence of a conversion disorder: it is a mode of speech that the patient has perfected over the years. Many aspects of her foreign accent are more outspoken in the 2003 speech sample as compared to equivalent speech output 8 years earlier.

On the basis of the considerations presented above, we feel that the speech mode in this patient is clearly distinct from what is typically observed in Foreign Accent Syndrome and should not be considered as another example of FAS. Rather, it exemplifies the existence of psychogenic foreign accent behaviour associated with conversion disorder. As such, these data do not contribute significantly to the discussion of whether or not Foreign Accent Syndrome is a truly organic disorder.

## Acknowledgments

We would like to thank Dr. J. Gurd from the Neuropsychology Research Unit, University Department of Clinical Neurology, Oxford, who was so kind to bring this patient to our attention. We very much appreciate the help of Guy De Pauw from the Centre of Dutch Language and Speech, Antwerp, who computed the Type/Token ratios and word complexity measures in the Dutch reference corpus. We also acknowledge the editorial assistance of Koen Meeussen.

## References

[1] A. Aaronson, *Clinical Voice Disorders*, New York: Thieme, 1990.

[2] A. Alekoumbides, R.A. Charter, T.G. Adkins and G.F. Seacat, The diagnosis of brain damage by the WAIS, WMS, and Reitan Battery utilizing standardized scores corrected for age and education, *International Journal of Clinical Neuropsychology* **9** (1987), 11–28.

[3] A. Ardila, M. Rosselli and O. Ardila, Foreign accent: an aphasic epiphenomenon? *Aphasiology* **2** (1988), 493–499.

[4] A.L. Benton, K. deS Hamsher, N.R. Varney and O. Spreen, *Contributions to Neuropsychological Assessment: A Clinical Manual*, New York: Oxford University Press, 1983.

[5] American Psychiatric Association, *Diagnostic and Statistical Manual of Mental Disorders, Fourth Edition*, Text Revision (DSM-IV-TR), Washington DC: American Psychiatric Association, 2000.

[6] A. Pick, Über Änderungen des Sprachecharakters als Begleiterscheinung aphasischer Störungen, *Zeitschrift für die gesamte Neurologie und Psychiatrie* **45** (1919), 230–241.

[7] *Army Individual Test Battery*, Manual of Directions and Scoring, Washington (DC): War Department, Adjutant General, 1944.

[8] C.J. Golden, *Stroop color and word test: a manual for clinical and experimental uses*, Chicago (IL): Stoelting, 1978.

[9] C.W. Whitty, Cortical dysarthria and dysprosody of speech, *Journal of Neurology, Neurosurgery and Psychiatry* **27** (1964), 507–510.

[10] D. Wechsler, *WAIS-R Manual*, New York: Psychological Corporation, 1981.

[11] D. Wechsler, *Wechsler Memory Scale-Revised manual*, San Antonio (TX): Psychological Corporation, 1991.

[12] E.J. Roth, K. Fink, L.R. Cherney and K.D. Hall, Revision to a previously learned foreign accent after stroke, *Archives of Physical and Medical Rehabilitation* **78** (1997), 550–552.

[13] E. Kaplan, H. Goodglass and S. Weintraub, *The Boston Naming Test*, Philadelphia: Lea & Febiger, 1983.

[14] E. Puttemans, Foreign Language Syndrome, Vrije Universiteit Brussel: Unpublished MA dissertation, 2005.

[15] G. Denes, J. Trumper, M. Maddalon and L. Romito, *Foreign Accent Syndrome: an Italian Case Study*, Proceedings of the XXth Congress of Phonetic Sciences, 1988, 662–664.

[16] G.H. Monrad-Krohn, Dysprosody or altered "melody of language", *Brain* **70** (1947), 405–415.

[17] G.M. Seliger, G.M. Abrams and A. Horton, Irish brogue after stroke, *Stroke: a Journal of Cerebral Circulation* **23** (1992), 1655–1656.

[18] H.A. Whitaker, Foreign accent syndrome, in: *Neuropsychology and Cognition*, (Vol. 1), series D, no. 9. R.N. Malatesha and L.C. Hartlage, eds, NATO Advanced Study Institute Series, The Hague: North Atlantic Treaty Organization, 1982, pp. 168–207.

[19] H.B. Schiff, M.P. Alexander, M.A. Naeser and A.M. Galaburda, Aphemia: clinical-anatomic correlations, *Archives of Neurology* **40** (1983), 720–727.

[20] H. Goodglass and E. Kaplan, *The Assessment of Aphasia and Related Disorders*, Philadelphia: Lea & Febiger, 1983.

[21] I. Moen, A case of the 'foreign-accent-syndrome', *Clinical Linguistics & Phonetics* **4** (1990), 295–302.

[22] J.C.L. Ingram, P.F. McCormack and M. Kennedy, Phonetic analysis of a case of foreign accent syndrome, *Journal of Phonetics* **20** (1992), 457–474.

[23] J. Dankovicova, J.M. Gurd, J.C. Marshall, J.S. Coleman, M.K.C. MacMahon, J. Stuart-Smith and A. Slater, Aspects of non-native pronunciation in a case of altered accent following stroke (foreign accent syndrome), *Clinical Linguistics and Phonetics* **15** (2001), 195–218.

[24] J.M. Gurd, N.J. Bessell, R.A.W. Bladon and J.M. Bamford, A case of foreign accent syndrome, with follow-up clinical, neuropsychological and phonetic descriptions, *Neuropsychologia* **26** (1988), 237–251.

[25] J.M. Gurd, J.S. Coleman, A. Costello and J.C. Marshall, Organic of functional? A new case of foreign accent syndrome, *Cortex* **37** (2001), 715–718.

[26] J.M. Nielson and M. McKeown, Dysprosody: report of two cases, *Bulletin of the Los Angeles Neurological Societies* **26** (1961), 157–159.

[27] J.R. Graham, *MMPI-2, Assessing personality and psychopathology*, (2nd ed.), New York: Oxford University Press, 1993.

[28] J. Van Borsel, L. Janssens and P. Santens, Foreign accent syndrome: A case report and some reflections, *Journal of Communication Disorders* **38** (2005), 421–429.

[29] J. Vanderlinden, W. Vandereycken, R. van Dyck and H. Vertommen, Dissociative experiences and traume in eating disorders, *International Journal of Eating Disorders* **13** (1993), 187–193.

[30] J. Verhoeven, G. De Pauw and H. Kloots, Speech rate in a pluricentric language situation: a comparison between Dutch in Belgium and the Netherlands, *Language and Speech* **47** (2004), 297–308.

[31] J. Verhoeven and P. Mariën, Prosodic characteristics of a case of Foreign Accent Syndrome, *Antwerp Papers in Linguistics* **100** (2002), 131–148.

[32] K.M. Kurowski, S.E. Blumstein and M. Alexander, The foreign accent syndrome: a reconsideration, *Brain and Language* **54** (1996), 1–25.

[33] M. Berthier, A. Ruiz, M. Massone, S.E. Starkstein and R.C. Leiguarda, Foreign accent syndrome: behavioural and anatomical findings in recovered and non-recovered patients, *Aphasiology* **5** (1991), 129–147.

[34] M. Critchley, Regional "accent", *Demotic Speech, and Aphasia, in Livre Jubilaire Docteur Ludo Van Bogaert*, Bruxelles: Les Editions Acta Medica Belgica, 1964, 182–191.

[35] M.D. Lezak, *Neuropsychological assessment*, New York: Oxford University Press, 1983.

[36] M. Folstein, S. Folstein and P. McHugh, Mini-Mental State: a practical method for grading the cognitive state of patients for the clinician, *Journal of Psychiatric Research* **12** (1975), 189–198.

[37] M.G. Cole and D. Dastoor, A new hierarchic approach to the measurement of dementia, *Psychosomatics* **28** (1987), 298–305.

[38] M. Moonis, J. Swearer, S. Bloomstein, K. Kurowski, R. Licho, P. Kramer, A. Mitchell and D.L. Osgood, Foreign accent syndrome following a closed-head injury: perfusion deficit on Single Photon Emission Tomography with normal Magnetic Resonance Imaging, Neuropsychiatry, *Neuropsychology and Behavioral Neurology* **9** (1996), 272–279.

[39] M. Trimble, *Somatoform disorders*, Cambridge: University Press, 2004.

[40] N. Miller and H. O'Sullivan, What makes foreign accent syndrome foreign? *ICPLA, International Clinical Phonetics and Linguistics Association*, 6th Annual Conference, Nijmegen, 13–15 October, poster presentation, 1997.

[41] N.R. Graff-Radford, W.E. Cooper, P.L. Colsher and A.R. Damaso, An unlearned foreign "accent" in a patient with aphasia, *Brain and Language* **28** (1986), 86–94.

[42] P.A. Osterrieth, Le test de copie d'une figure complexe, *Archives of Psychology* **30** (1944), 205–353.

[43] P. Graetz, R. De Bleser and K. Willmes, *De Akense Afasie Test*, Lisse: Swets & Zeitlinger, 1992.

[44] P. Mariën, E. Mampaey, A. Vervaet, J. Saerens and P.P. De Deyn, Normative data for the Boston Naming Test in native Dutch-speaking Belgian elderly, *Brain and Language* **65** (1998), 447–467.

[45] R.C. Oldfield, The assessment and analysis of handedness: the Edinburgh inventory, *Neuropsychologia* **9** (1971), 97–113.

[46] R.R. Reeves and J.W. Norton, Foreign accent-like syndrome during psychotic exacerbations, *Neuropsychiatry, Neuropsychology and Behavioral Neurology* **14** (2001), 135-138.

[47] S.E. Blumstein, M.P. Alexander, J.H. Ryalls, W. Katz and B. Dworedzky, On the nature of the foreign accent syndrome: a case study, *Brain and Language* **31** (1987), 215–244.

[48] S.R. Hathaway and J.C. McKinley, *The Minnesota Multiphasic Personality Schedule* (*revised*), Minneapolis, MN: University of Minnesota Press, 1943.

[49] T. Coughlan, S. Lawson and D. O'Neill, French without tears? foreign accent syndrome, *Journal of the Royal Society of Medicine* **97** (2004), 242–243.

[50] T.J. Carbary, J.P. Patterson and P.J. Snyder, Foreign accent syndrome following a catastrophic second injury: MRI correlates, linguistic and voice pattern analyses, *Brain and Cognition* **43** (2000), 78–85.

[51] W.G. Rosen, R.C. Mohs and K.L. Davis, A new rating scale for Alzheimer's disease, *American Journal of Psychiatry* **141** (1984), 1356–1364.

[52] Y. Takayama, M. Sugishita, T. Kido, M. Ogawa and I. Akiguchi, A case of foreign accent syndrome without aphasia caused by a lesion of the left precentral gyrus, *Neurology* **43** (1993), 1361–1363.
